# Effect of pathologic fractures on survival in multiple myeloma patients: a case control study

**DOI:** 10.1186/1756-9966-27-11

**Published:** 2008-06-10

**Authors:** Mehmet Sonmez, Tulin Akagun, Murat Topbas, Umit Cobanoglu, Bircan Sonmez, Mustafa Yilmaz, Ercument Ovali, Serdar Bedii Omay

**Affiliations:** 1 Department of Haematology, Karadeniz Technical University, School of Medicine, Trabzon, Turkey; 2 Department of Internal Medicine, Karadeniz Technical University, School of Medicine, Trabzon, Turkey; 3 Department of Public Health, Karadeniz Technical University, School of Medicine, Trabzon, Turkey; 4 Department of Pathology, Karadeniz Technical University, School of Medicine, Trabzon, Turkey; 5 Department of Nuclear Medicine, Karadeniz Technical University, School of Medicine, Trabzon, Turkey

## Abstract

**Background:**

Multiple Myeloma (MM) is a B cell neoplasm characterized by the clonal proliferation of plasma cells. Skeletal complications are found in up to 80% of myeloma patients at presentation and are major cause of morbidity.

**Methods:**

49 patients were enrolled with MM admitted to Black Sea Technical University Hospital between 2002–2005. Pathologic fractures (PFs) were determined and the patients with or without PF were followed up minumum 3 years for survival analysis.

**Results:**

PF was observed in 24 patients (49%) and not observed in 25 patients (51%). The risk of death was increased in the patients with PF compared with patients who had no fractures. While overall survival was 17.6 months in the patients with PFs, it was 57.3 months in the patients with no PFs.

**Conclusion:**

These findings suggest that PFs may induce reduced survival and increased mortality in the MM patients, however, larger sample size is essential to draw clearer conclusions added to these data.

## Background

MM is a B cell neoplasm characterized by the clonal proliferation of plasma cells within the bone marrow. Skeletal complications including enhanced bone loss associated with diffuse osteopenia, focal lytic lesions, PFs, hypercalcemia, and bone pain are found in up to 80% of myeloma patients at presentation and are major causes of morbidity. Increased osteoclastic activity combined with inhibited osteoblastic activity contribute to the skeletal complications. The increased bone turnover has been characterized as an important facilitator of proliferation and tumor cell survival in MM [1-3].

PFs are important healthcare concern in patients with MM. They may cause severe bone pain, limit mobility, and require surgery and hospitalization for treatment. Thus, fractures can interfere with functional independence and may shorten survival. Although a relationship between PFs and survival in patients with MM has been reported with clinical observations, there are limited data on correlation pattern [4].

In the present study, we planned to determine the impact of PFs on survival in patients with MM.

## Methods

49 patients were enrolled with MM admitted to Black Sea Technical University Hospital between 2002–2005. All patients had myeloma diagnosis and treatment criteria. PFs were determined with radiography, scintigraphy, and Magnetic Resonance Imaging (MRI), if radiography is insufficient for diagnosis or patients having no visuable lesions in the radiography are symptomatic. Treatment was begun to all patients and continued to provide remission and plateau phase. Treatment options including melphalan, prednisolone, vincristine, dexamethasone, adriamycine, cyclophosphamide, thalidomide, bortezomib, bisphosphonates, autologous transplantation were planned case by case. Due to myeloma treatment guideline, treatment modalities were arranged as first, second, and third line. Melphalan-Prednisolone (MP) and Vincristine-Adriamycine-Dexamethasone (VAD) were planned as first line treatment; other regimens were planned as second and third line. The patients with or without PF were followed up minumum 3 years for survival analysis.

### Statistical Analysis

Survival was studied from the date of diagnosis to the last contact with the patient. Survival curves were calculated using the Kaplan-Meier method and statistical comparisons were performed by the log-rank test. The relationship between predictor factors and survival was examined using Cox's regression analysis. Quantitative data were analysed using the chi-square tests. P values <0.05 were considered to be significant.

## Results

**Figure 1:**
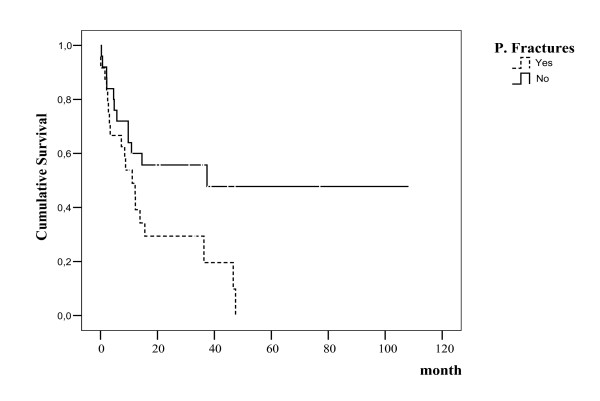
Overall survival in patients with MM.

Of the 49 patients, 29 were male and 20 were female. The mean age was 63.3 ± 11.6 (min-max, 40–80). 19 patients were Immunoglobuline (Ig) G, 16 patients were IgA, 7 patients were light chain, and 7 patients were nonsecretory type. 36 patients were stage 3, 10 patients were stage 2, and 3 patients were stage 1 according to Durie-Salmon classification. The laboratory values of the patients are presented in Table 1. 41 patients (83.7%) had lytic lesions and 8 patients (16.3%) had no lytic lesions. PF was observed in 24 patients (49%) and not observed in 25 patients (51%). PFs were defined vertebral and nonvertebral. 17 patients had vertebral fractures and 7 patients had fractures in other sides including femur, humerus, ribs, and bisphosphonate therapy was administered to all patients with both zoledronic acid and pamidronate. 9 patients required surgical treatment and 10 patients required radiotherapy. Response to first line treatment was determined in 19% of the patients with PF. The risk of death was increased in the patients with PF compared with patients who had no fractures (p = 0.03). While overall survival was 17.6 months in the patients with PF, it was 57.3 months in the patients with no PF (according to Kaplan Meire test, log rank p = 0.03) (Figure 1). In the Cox regresion analysis, PFs were a significant predictor of decreased survival (OR: 2.62; 95% CI:1.12–6.10, p = 0.02). The effect on survival is independent compared to other prognostic parameters including hemoglobin, calcium, stage, albumine, α_2 _microglobulin, and lytic lesions.

**Table 1:** Laboratory parametes of patients

n = 49	Mean	Minimum-Maximum
Hb (g/dL)	9.08 ± 2.18	5–13.3
Creatinine (g/dL)	1.9 ± 1.6	0.5–6.6
B_2 _microglobulin (mg/dL)	1.54 ± 1.93	0.2–7.7
LDH (U/L)	416.9 ± 226.2	112–1378
Albumin (g/dL)	3.2 ± 0.76	1.8–5.1
Calcium (mg/dL)	10.36 ± 1.89	7.7–15.3

## Discussion

PFs occur in approximately 40% of patients with multiple myeloma [5]. More recent studies with the addition of MRI assessment show a much higher proportion of patients actually have fractures at presentation [6]. PFs result not only from the direct deposits of myeloma cells within the bone, but also from the release of soluble factors by both the tumor and the bone microenvironment, resulting in the stimulation of osteoclast activity and bone resorption [7]. Osteoclast-activating factors (OAFs) and receptor activator of nuclear factor-κB (RANK) ligand (RANKL)-osteoprotegerin-RANK system have provided a better understanding of myeloma bone disease in molecular level. Myeloma cells adhere to the stromal cells and induce secretion of OAFs including interleukin (IL)-6, IL-1, tumor necrosis factor (TNF), IL-11, macrophage inflammatory protein-1α (MIP-1α), hepatocyte growth factor (HGF), parathyroid hormone-related peptide (PTHrP), and others. OAFs increase expression of RANKL on the marrow stromal cell surface. RANKL binds to the RANK receptor on osteoclast precursors and triggers osteoclast differentiation and activation. This interplay among the activated osteoclast, stromal cells and OAFs in the bone marrow microenvironment, in turn, stimulates MM cells growth. The activity of RANKL can be blocked by osteoprotegerin, preventing the interaction between RANK and RANKL. Also, inhibition of osteoblast activity plays a role in the pathogenesis of MM bone disease. Dickkopf (DKK)-1, an inhibitor of Wnt signaling secreted by MM cells, causes a hindered osteoblast differentiation and activity. In addition to this mechanism, malignant plasma cells are able to induce osteoblast apoptosis [8-10].

PFs have been associated with a 23–32% increased risk of mortality in prostate cancer, breast cancer, and MM [11]. Several factors may affect the situation: for example, loss of mobility and functional independence, increased risk of deep vein thrombosis, surgery for fracture, and biologic behavior of disease. In the recently published metaanalysis, it was concluded that PFs correlate with reduced survival in patients with malignant bone disease [4]. Our study similar to this metaanalysis, showed that MM patients with PF have reduced survival and increased mortality probably with biologic behavior of MM. Because, no relationship between PFs and other activity parameters of MM was observed. It is an independent factor for survival and may be related to the pathogenesis of bone disease. As viewed in the literature, use of bisphosphonates, which involves the inhibition of osteoclast-mediated bone resorption, is together with demonstrated improved survival and reduced PFs. A decreasing marker of bone resorption, N-telopeptide of type I collagen, in the bone metastases patients, treated with zoledronic acid, associated with increased overall survival [11-13]. All data have showed that myeloma bone disease has a different effect in the prognosis of MM.

The most common site of fractures is in the spine (55%–70%) especially in the lower thoracic or lumber vertabral bodies. Vertebral compression fractures had increased nearly a three-fold compared to a healthy person [3,14]. In our patients, vertebral fractures frequency were higher compared to other fracture sides. Older individuals with vertebral fractures have an increased risk of mortality. Kado et al. reported that women with vertebral fractures had an age-adjusted 32% increased risk of mortality compared with women without incident vertebral fractures [15]. As a whole, vertebral fractures independently plus MM-related vertebral fracture incidence contribute to the higher morbidity rate in MM patients.

## Conclusion

These findings suggest that PFs may cause reduced survival and increased mortality in the MM patients, however, larger sample size is essential to draw clearer conclusions added to these data.

## Competing interests

The authors declare that they have no competing interests.

## Authors' contributions

MS has made substantial contributions to conception and design, TA has made substantial contribution to acquisition of the data, MT has performed statistical analysis, UC has performed the diagnostical pathological analysis, BS has performed diagnostical and therapeutic response analysis by nuclear medicine methods, MY has made substantial contribution to acquisition of data, EO has made the interpretation of data, SBO has given the final approval of the version.
